# CImbinator: a web-based tool for drug synergy analysis in small- and large-scale datasets

**DOI:** 10.1093/bioinformatics/btx161

**Published:** 2017-03-19

**Authors:** Åsmund Flobak, Miguel Vazquez, Astrid Lægreid, Alfonso Valencia

**Affiliations:** 1 Department of Cancer Research and Molecular Medicine, Norwegian University of Science and Technology (NTNU), Trondheim, Norway; 2 Structural Computational Biology Group, Structural Biology and Biocomputing Programme, CNIO (Spanish National Cancer Research Centre), Madrid, Spain

## Abstract

**Motivation:**

Drug synergies are sought to identify combinations of drugs particularly beneficial. User-friendly software solutions that can assist analysis of large-scale datasets are required.

**Results:**

CImbinator is a web-service that can aid in batch-wise and in-depth analyzes of data from small-scale and large-scale drug combination screens. CImbinator offers to quantify drug combination effects, using both the commonly employed median effect equation, as well as advanced experimental mathematical models describing dose response relationships.

**Availability and Implementation:**

CImbinator is written in Ruby and R. It uses the R package drc for advanced drug response modeling. CImbinator is available at http://cimbinator.bioinfo.cnio.es, the source-code is open and available at https://github.com/Rbbt-Workflows/combination_index. A Docker image is also available at https://hub.docker.com/r/mikisvaz/rbbt-ci_mbinator/.

**Supplementary information:**

Supplementary data are available at *Bioinformatics* online.

## 1 Introduction

The discovery of growth inhibiting drug synergies is important because they allow for a lower dosage of each drug while preserving effects. Drug synergies can thus be exploited to increase drug treatment responses to levels that are normally limited by the toxicity and side effects of each drug. These properties warrant large drug combination screens to discover new synergies (Axelrod *et al.*, 2013; Crystal *et al.*, 2014; Mathews Griner *et al.*, 2014). While currently available software have typically been devised for small scale experiments, the increase in sizes of drug panels demand software solutions designed to batch-wise analyze large-scale combination screen data. Furthermore, user-friendly designs are required to accommodate both biologists and bioinformaticians studying drug synergy.

We here present CImbinator, a software tool that offers substantial advance in assisting assessment of drug response effects and synergies from small and large-scale experiments. CImbinator provides an intuitive user interface to upload data for large datasets from a user-supplied datafile. Data from small datasets can also be entered manually. CImbinator enables the user to quickly orient in a large experimental search space through its display in a single web window of color-coded synergy assessments for all drug combinations describing each dose tested. In-depth details of each combination are easily accessible by selecting the drug combination, including dose–response plots, combination effect plots, combination indices and confidence bands.

**Fig. 1:**
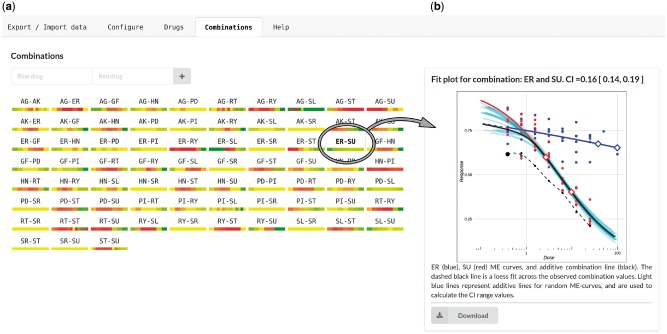
An intuitive user inter-face presents the drug synergy assessment for each drug combination in a birds eye overview matrix (**a**), with color-coded bars for each drug combination to allow for a quick evaluation of synergies (green = synergy, yellow = additivity, red = antagonism). Individual drug combination responses can be inspected more closely with dose–response relationships for each single drug and the drug combination (**b**), including combination indexes as confidence bands. See Supplementary Material and online help-section for further information

CImbinator assesses drug combination effects based on the ‘Loewe additivity model’ (Loewe, 1953), which is one of the most popular approaches among mathematical models introduced to analyze drug combination effects. Loewe additivity relates the doses of each drug in a combination that elicit a given effect to the doses of each individual drugthat would be required to achieve the same effect level in either single drug formulation (reviewed in Greco *et al.*, 1995). We estimate effect levels for any dose based on either the median effect model (Chou, 2010), or based on advanced three or four-parametric dose response relationships. Importantly, our extension of the drug synergy analysis with a stochastic procedure enables CImbinator to estimate confidence intervals for the combination indices. The online application contains sample data and an interactive step-by-step tutorial to ease user experience. The source code is freely available and allows to deploy the web page locally or use the functionalities from the command-line or from Ruby scripts (Fig. 1).

## 2 Methods and functionality

CImbinator provides:

- a batch analysis tool, combined with results presented in a ‘birds eye view’ to facilitate investigations of large datasets
- quick access to details of drug combination results
- alternative dose-relationship models (least squares fit, log-logistic LL.2-5 fits)
- uncertainty estimates for predicted additive responses, based on uncertainties in single drug dose–response relationships
- export of publication-ready graphics (SVG format)
- an open source software analysis tool for drug synergy assessment
- web-server backend methods exposed for programmatic access
- several example datasets

CImbinator provides several approaches to determine dose–response curve parameters:

1. a least squares method as originally proposed by Chou, and used in the commercial software packages CompuSyn and CalcuSyn, and
2. several advanced alternative log-logistic dose–response models (LL.2-LL.5) from the R package drc, which have different assumptions on the shape of the drug response curve and which can potentially provide a better fit when a dose–response relationship cannot be approximated by the median effect equation.

For two drugs the additive effect (i.e. assuming no interaction among the drugs with respect to the measured response) is displayed together with the drug response curve of each drug. When possible, random additive contours are computed by estimating the median effect points from the predictive distribution of the single-drug response fits (95% confidence; for least squares or LL.*). These random additive lines serve as confidence bands for the additive line, and are used to compute the CI value intervals.

## 3 Use-cases for CImbinator

To demonstrate some of the capabilities of CImbinator, we have uploaded the publicly available datasets from Miller *et al.* (2013), Haagensen *et al.* (2012) and Szwajda *et al.* (2015). See the Supplementary Material for these use-cases.

## 4 Discussion and conclusion

The exponentially increasing search space of drug combination screens calls for novel software solutions to efficiently identify and characterize drug synergies from experimental data is dearly needed. Currently available software, including Combenefit (Di Veroli *et al.*, 2016), CompuSyn or Chalice (http://cwr.horizondiscovery.com/) often require either extensive computer skills, particular operating systems, or are designed for low-throughput experiments (see Supplementary Table S1 for a comparison of CImbinator and other tools). We tackle these obstacles by providing a user-friendly open-source online software application that accepts batch-wise input from large experiments and an intuitive user-interface that enables novel analysis approaches to facilitate drug combination screening.

## Supplementary Material

Supplementary Data
