# Supplementary material for: CImbinator: a web-based tool for drug synergy analysis in small- and large-scale datasets
Source: Bioinformatics. 2017 Mar 19;33(15):2410–2. doi: 10.1093/bioinformatics/btx161 (PMC5860113; doi:10.1093/bioinformatics/btx161)
Supplement: Supplementary Data [file btx161_supplementary_material.docx]

Supplementary Material

# Tutorial

## Quick introduction to CImbinator in three steps

**Step 1: Importing data.** The CImbinator web-page (<http://cimbinator.bioinfo.cnio.es/>) can be accessed through a standard modern web browser. At the top of the web page the user can access different parts of the service by selecting the relevant tab from the set of available tabs: *Export/Import data*, *Configure*, *Drugs*, *Combinations* and *Help*. Typically one would start by importing data, which can be done by selecting *Export/Import data* (see Fig. S1). The import of data can be done either by loading a data file (see Supplementary Material chapter 4), or by pasting the data as text into the textbox. To import one of the example datasets provided by the web page select the *Miller dataset* and next click *Submit*.


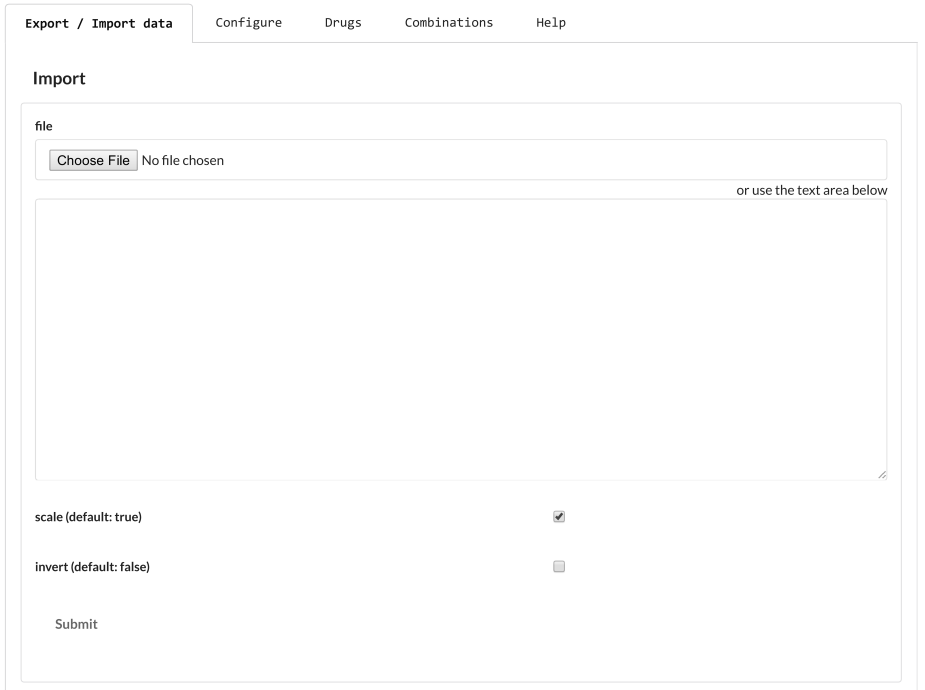


**Fig. S1: Import and Export data to and from CImbinator**. Data can be imported either from file by selecting 'Choose File' or by pasting text input to the textbox. Data can also be exported to either Excel files or tab separated files.

**Step 2: Configuring drug combination response analysis.** After data has been imported and submitted the analysis can be configured by accessing the second tab *Configure*. In the dropdown menu *Model type* the user can choose a relevant drug combination model that is used to calculate the expected combination response, e.g. *Loewe additivity* (see Fig. S2). If the user next selects *Analyze all in batch* the web server will issue a batch analysis to report on drug combination effects for all drug combinations submitted.


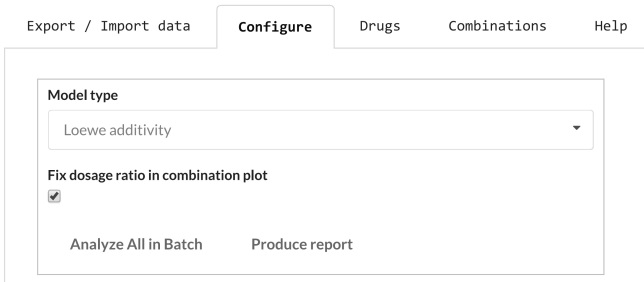


**Fig. S2. Configuring the analysis.** The user can choose one of several model types that is used to calculate the expectation combination response. This expectation is used to quantify synergy, which is the excess between the observed effect to the expected effect.

**Step 3: Study drug responses.** After submitting data and configuring the analysis CImbinator allows exploration of single and combination drug responses. The single drug responses can be found in the tab *Drugs.* This tab also allows for manual editing of existing data and entering of new data. To add a drug to the dataset input the drug name followed by the selecting the plus sign, and then one can add measurements by inputting *Dose* and *Response* for the new drug. Fig. S3 shows an example of what this tab can look like for the Miller dataset, and Fig. S4 shows an example of the drug combination overview matrix for the Miller dataset.


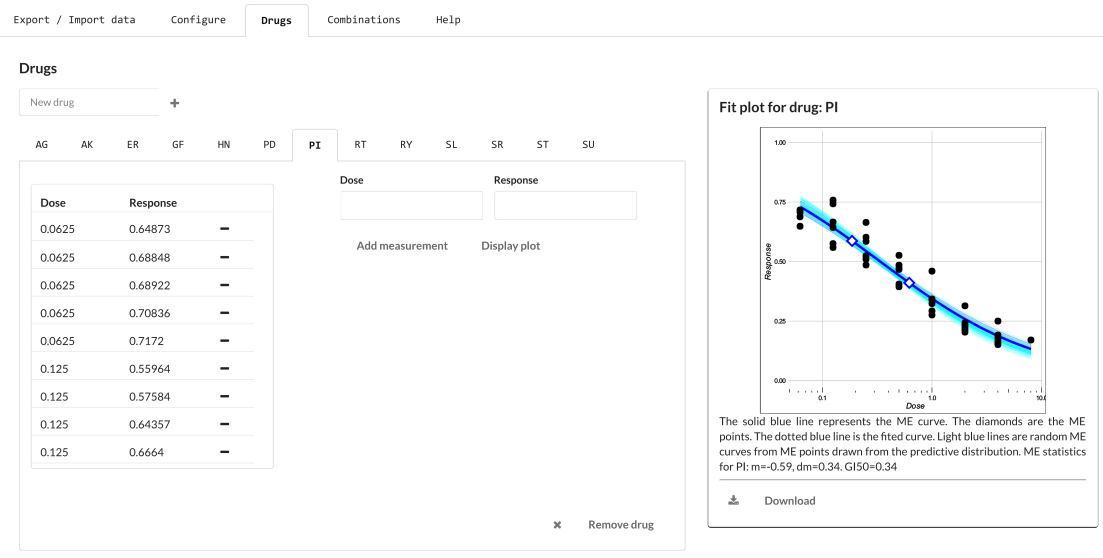
**Fig. S3. Single drug responses.** In the *Drugs* tab the single drug dose response fits can be displayed by selecting *Display plot*. New drugs and observations can be added manually by typing in the name of a new drug followed by clicking the plus sign, or by adding *Dose* and *Response* data for the new drug. Existing drugs can be removed by selecting *Remove drug*, and observations can be removed by clicking the minus sign in the rightmost column in the dose-response table.


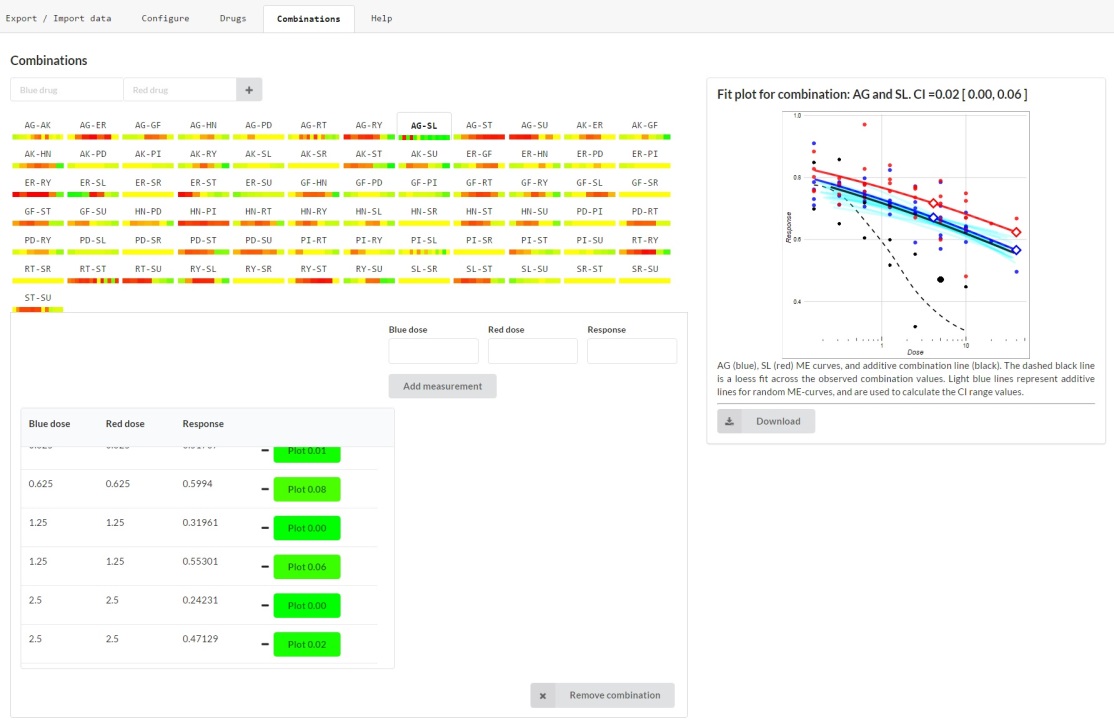


**Fig. S4. Drug combination responses.** Drug combation response matrix (‘birds eye overview’) for the Miller dataset, and detailed dose response plot for the drug combination AG-SL. The matrix displays a color-coded bar above each drug pair, which is a graded synergy assessment across increasing doses from left to right (green = synergy, yellow = additive, red = antagonism). Example: If a bar is yellow at the left end and green at the right end it means this drug combination acts additively at lower doses and synergistically at higher doses. The detailed dose response plot is displayed by first selecting a drug pair in the matrix, and then selecting *Plot* in the dose response table. The *Plot* button also displays the quantified synergy score as a number, e.g. Combination Index (CI) values for Loewe additivity analyses. The detailed dose response plot also gives confidence scores for the CI values. In the example of drugs AG and SL the CI value is 0.02, with confidence bands indicating the CI value to lie between 0.00 and 0.06.

## Miller dataset

The Miller dataset was produced to assess drug synergies in a panel of 105 combinations targeting a dedifferentiated liposarcoma cell line (Miller et al., 2013). The dataset can be loaded by performing the following steps:

- Click on *Miller dataset* in the *Export/Import data* tab. The dataset will be loaded to the *Import data* textbox.
- Click *Submit.*
- Select model type under the *Configure* tab, e.g. *Loewe additivity.*
- Click *Analyze all in batch.*

Fig. 1 in the main text and Fig. S3 in the Supplementary Material above show the analysis of the Miller dataset. Each color bar in the overview depicts the synergy assessment at increasing doses from left to right (green for synergy, yellow for additivity, red for antagonism). Inspection of the CImbinator visualization of CI values across all doses indicates that some of the drug combinations, identified by Miller et al. (Miller et al., 2013) to be synergistic at GI50 doses, are mainly synergistic at GI50-centered dose ranges, while they can act additively at other dose ranges. This applies e.g. to combined targeting of AKT and MEK (AK-SL) where the synergy is mostly displayed at GI50-centered doses, while the synergy of targeting PDGFR and MEK (PD-SL) is consistent across all doses tested.

Notably, CImbinator can compute combination responses for all 91 drug combinations, when configured to use least squares fitting. Thus, CImbinator analyses indicate that IGF1R and ERK inhibition (AG-SL) act strongly synergistic for most concentrations. The effect of the IGF1R and ERK (HN-SL) combination was reported not to be computable by Miller et al.

CImbinator allows detailed inspection of each drug combination experiment, with single drug responses, expected additive behavior, and observed combination effects (see main text Fig 1B, and see Supplementary Fig. S5 for an explanation of main text Fig 1B).

## The Haagensen dataset

The Haagensen dataset (Haagensen et al., 2012) compares combinations of MEK inhibitors with either specific PI3K inhibitors or dual mTOR/PI3K inhibitors. The dataset can be loaded by performing the following steps:

- Click on *Haagensen dataset* in the *Export/Import data* tab. The dataset will be loaded to the *Import data* textbox.
- Ensure that *Scale* is checked (some effects are reported to be negative; CImbinator will scale all responses to lie between 0 and 1, rendering all observations computable).
- Click *Submit.*
- Select model type under the *Configure* tab, e.g. *Loewe additivity.*
- Click *Analyze all in batch.*

A batch analysis of the Haagensen dataset quickly reveals the main finding from their publication, that MEK inhibitors (PD0325901 or AZD6244) act synergistically stronger together with a specific PI3K inhibitor (GDC9041) than a dual PI3K/mTOR inhibitor (NVP-BEZ235) (visualized upon analysis of the Haagensen dataset by the more saturated green bars for the MEK-PI3K combinations than the MEK-PI3K/mTOR combinations).

## The Szwajda dataset

The Szwajda dataset (Szwajda *et al.*, 2015) provides experimental validation data for top predictions of drug synergies for a subset of 40 kinase inhibitors evaluated for the use in breast cancer. The dataset can be loaded by performing the following steps:

- Click on *Szwajda dataset* in the *Export/Import data* tab. The dataset will be loaded to the *Import data* textbox.
- Ensure that *Scale* and *Invert* are checked (this dataset reports ‘percent inhbition’, so invert is needed to convert to viability data).
- Click *Submit.*
- Select model type under the *Configure* tab, e.g. *Bliss independence.*
- Click *Analyze all in batch.*

## Overview of parameters that can be adjusted

**Export/Import data tab:**

**Scale** When checked *Scale* will ensure that all observations are scaled to be in the range 0..1

**Invert** When checked *Invert* will ensure that data is inverted, i.e. observations close to one will be close to zero, and vice versa. For Loewe additivity analyses the inversion doesn’t affect the computation, but for Bliss independence and Highest Single Agent (HSA) the inversion might be needed to convert data to ‘viability’ data, i.e. data where high doses corresponds to low observations numerically.

**Configure tab:**

**Model type** Model type is used to assign a drug combination expectation model to the analysis. CImbinator supports three models: Loewe additivy, Bliss independence and Highest Single Agent.

For the Loewe additivity CImbinator offers the option to use three, four or five-parametric dose response curves, in addition to the traditional two-parametric curve used to compute CI values (Chou and Talalay 1984). When selecting these advanced dose response curves the user is offered to choose an effect range that is used to estimate a two-parametric dose response curve, which is next used for estimating CI values. Alternatively, the user can check *Compute CI directly from dose-response fit* to use the advanced dose response curves directly when computing CI values, but this approach is more computationally intensive and takes longer than using the traditional two-parametric dose response curve for CI values.

**Fix dosage ratio** When checked the doses for single drugs in combination plots will be shifted to the right, so they align with the dose used in a combination. Example: If drug A and B is tested in a combination of doses 0.3 and 0.5 respectively, the “combination dose” is 0.3+0.5=0.8, and the response for the single drugs at doses 0.3 and 0.5 will be aligned with the combination dose.

# Interpretation of detailed CImbinator dose-response combination plots


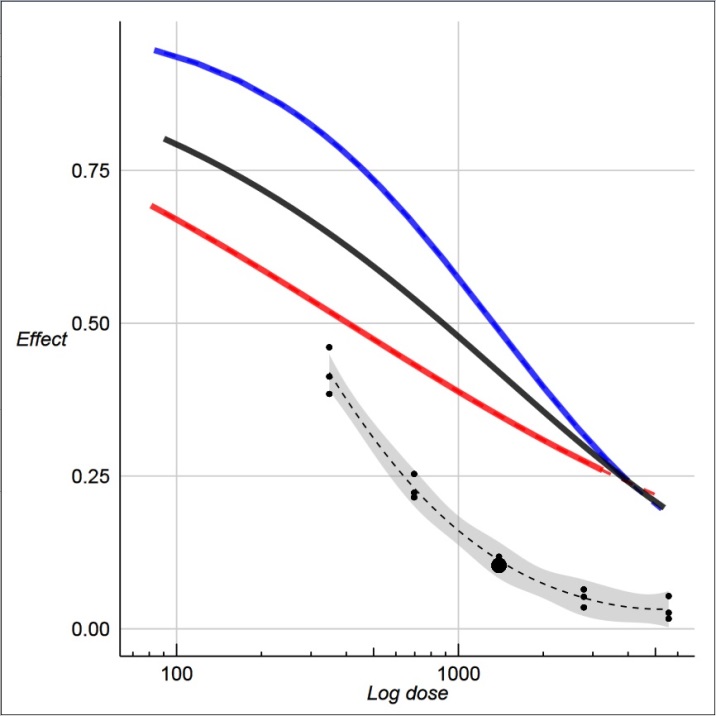
**Fig. S5. Toy drug combination experiment visualized by CImbinator.** The dose-response curve of each drug A (red) and B (blue) are plotted, and the theoretical additive line (black) is computed, here based on Loewe additivity (equation 1 in Chapter 6 of the Supplementary Material). Observed combination responses are plotted as dot(s) (black) and can be compared to the expected additive line. If the effect is more profound than the additive line this suggests the drugs act synergistically, and conversely if the effect is weaker the drugs act antagonistically.

# Availability

This software is free to use and released with an open-source licence.

**Source code**

The source code for this software is available at <https://github.com/Rbbt-Workflows/combination_index>. It is programmed in Ruby and uses R to produce the plots. The code is organized as an Rbbt (<http://mikisvaz.github.io/rbbt/>) workflow. The Rbbt workflow is called CombinationIndex (CImbinator is just the name for the web-app and Docker image)

**Installation**

You will need a working Ruby installation, such as those typically provided by Linux distributions. In addition to that you will need to have the Rbbt gems, and a working R installation with some packages installed.

To install Rbbt you can consult the getting started documentation (<http://mikisvaz.github.io/rbbt/tutorial/getting_started/>). The R packages you need to install are best consulted in the CImbinator Dockerfile (<https://github.com/Rbbt-Images/rbbt-docker-CImbinator/blob/master/Dockerfile>).

In fact, you may also use the Docker image mikisvaz/rbbt-ci_mbinator (<https://hub.docker.com/r/mikisvaz/rbbt-ci_mbinator/>).

**Starting a server**

There are two basic ways to use CImbinator: through the web interface or through the command line. You may use this web interface or you might start your own server on a local install doing:

rbbt workflow server CombinationIndex -e production -p 2887

Or, using Docker:

docker run -ti -p 127.0.0.1:2887:2887 -e DISPLAY=$DISPLAY mikisvaz/rbbt-ci_mbinator

You can now access the server http://localhost:2887/. Allow a couple of minutes on the first visit for the JavaScript and CSS assets to be prepared.

**Command-line use**

You may issue analysis jobs directly from the command-line. All the functionalities are used through the rbbt command. See the Rbbt documentation (<http://mikisvaz.github.io/rbbt/>) for more information on how to use it. Here are some examples:

See the available functionalities:

rbbt workflow task CombinationIndex

Check the parameters for any of the functionalities

rbbt workflow task CombinationIndex report -h

Note that when using any of the report functionalities the file needs to be a TSV file in any of the two collapsed formats (see below).

The result files of any of the tasks will be saved and will be re-used appropriately; for instance when single drug dose response fits are reused across combinations that feature them. To view the result file you can use the --printpath flag. Otherwise, the result is printed into STDOUT.

rbbt workflow task CombinationIndex report --file=~/share/data/CI/CI-mbinator-export.Miller.tsv --model_type=bliss --fix_ratio --printpath

When using any of the report features all the plots produced by the single combination evaluations are collected into a directory. You can find this directory alongside the result file, with a .files extension.

If you are using the docker image (<https://hub.docker.com/r/mikisvaz/rbbt-ci_mbinator/>) instead of the local installation you can just prepend docker run -ti -e DISPLAY=$DISPLAY mikisvaz/rbbt-ci_mbinator to any of the commands. Note of course that, as usual with Docker, any files passed to the commands must be available to the docker image, specifically when using the report methods, so you might need to mount a local directory into docker to make them available.

# Data formats

CImbinator requires dose-response data for single drugs and for drug combinations. This information can be imported from a file or entered manually.

**Import / Export**

For the convenience of the user, CImbinator supports two file types for importing and exporting data: Tab Separated Value files (TSV) and Excel spreadsheets (XSL or XSLX). Information in these files can also be laid out in several ways: expanded, collapsed columns and collapsed.

| **Drug** | **Second drug** | **Drug dose** | **Sencond drug dose** | **Response** |
| --- | --- | --- | --- | --- |
| BEZ235 |  | 5.25 |  | 0.84818 |
| BEZ235 |  | 21 |  | 0.44215 |
| AZD6244 |  | 78 |  | 0.679 |
| AZD6244 |  | 312 |  | 0.45525 |
| BEZ235 | AZD6244 | 5.25 | 78 | 0.60587 |
| BEZ235 | AZD6244 | 21 | 312 | 0.22932 |

*Example of expanded format*

| **Treatment** | **Dose** | **Response** |
| --- | --- | --- |
| BEZ235 | 5.25 | 0.84818 |
| BEZ235 | 21 | 0.44215 |
| AZD6244 | 78 | 0.679 |
| AZD6244 | 312 | 0.45525 |
| BEZ235-AZD6244 | 5.25-78 | 0.60587 |
| BEZ235-AZD6244 | 21-312 | 0.22932 |

*Example of collapsed column format*

| **Treatment** | **Dose** | **Response** |
| --- | --- | --- |
| BEZ235 | 5.25\|21 | 0.84818\|0.44215 |
| AZD6244 | 78\|312 | 0.679\|0.45525 |
| BEZ235-AZD6244 | 5.25-78\|21-312 | 0.60587\|0.22932 |

*Example of collapsed format*

To see some examples you can load any of the sample datasets provided in the *Export/Import data* section and export them to any of these three layouts in either TSV or Excel files. When importing the data from files the column header row must be present but the text of the headers is ignored, all that matters is the order of the columns. On TSV files or when inputting data in the textbox the header line must start with a pound character #.

Once the data is imported you may open the tabs *Drugs* and *Combinations* to examine the data and produce the plots. The tab *Configure* allows you to specify the details of how these are produced, as well as issue batch analyses.

The data loaded in the *Drugs* and *Combinations* tabs is saved on your browsers memory, and restored whenever the page is loaded. You may empty it using the *Reset* button from the *Export/Import data tab*, or you might introduce your own data points manually.

**Manual Input**

From the *Drugs* and *Combinations* tabs you might input new data-points manually. This functionality can be used to extend or amend data imported from a file or to input a new dataset by hand. Remember that your changes will be saved on the browser memory, but you can also export them to file to restore them at a later time.

# Comparison of CImbinator to other software solutions that offer combination data analysis

The CImbinator software offers a web-based service to analyse data from drug combination studies, providing for an open-source platform that employs several of the most commonly used drug combination expectation response models (see Table S1 for an overview).

|  | CImbinator | Combenefit | CompuSyn | Excel | Chalice |
| --- | --- | --- | --- | --- | --- |
| Source code | Yes | Yes | No | - | No |
| Open source | Yes | Yes (MIT) | No | - | No |
| Web interface | Yes | No | No | No | No |
| Synergy models | Loewe, Bliss, HSA | Loewe, Bliss, HSA | Loewe | - | Loewe, Bliss, HSA |
| Data input | File, Manual | File | Manual | Manual | File |
| HTS | Yes | Yes | No | - | Yes |
| Replicates | Yes | Yes | No | - | Yes |
| Dose-response fits | 2, 3, 4 or 5 params | 3 params | 2 params | - | 3 params |
| Experimental design | Ray | Matrix | Ray | - | Matrix, Ray |

**Supplementary Table S1. Overview of software for drug combination response analysis.** The table lists features of several available software packages to assist in drug combination response analsysis. HSA = highest single agent.

# Approach to drug combination response analyses

## Loewe additivity

We follow a two-step process in our analysis of each combination response: (1) characterize the individual dose-response curves for each drug, and (2) use this information to calculate CI values for the combination doses. CImbinator offers several options on how to perform these steps.

At the core of synergy assessment is the combination index (CI) equation,

$$CI= \frac{\left( D \right)_{1}}{\left( D_{x} \right)_{1}}+\frac{\left( D \right)_{2}}{\left( D_{x} \right)_{2}}$$

1

where (D)_1_ is the dose of drug 1 in the combination, and (D_x_)_1_ is the dose of drug 1 required to elicit an x percent effect alone (similarly for (D)_2_/(D_x_)_2_). The CI of any drug combination can then be quantified: CI values = 1 indicates additivity, whereas a CI < 1 indicates synergy and a CI > 1 indicates antagonism.

To compute the parameters (Dx)_1_ and (Dx)_2_ our default approach relies on the popular median effect equation (Chou and Talalay, 1984),

$$\frac{f_{a}}{f_{u}}=\left( \frac{D}{D_{m}} \right)^{m}$$

2

where *f_a_* and *f_u_* are the fraction affected and fraction unaffected, respectively, *D* is the dose, *D_m_* is the dose eliciting a median effect (e.g. GI50), and *m* is a coefficient describing the sigmoidality of the dose-response curve (see Supporting information).

#### Dose response fit and median effect statistics

Following a similar approach deployed in the commercial software application CompuSyn, our default approach characterizes the dose-response curves for each individual drug by fitting a least squares linear model to the median effect equation (equation 2). From this linear model we compute the values for the ‘median effect statistics’ *m* and *D_m_* (ME statistics). Note that the median effect equation assumes the drug response range to go from 0 to 1.

The ME statistics derive from the ME points using two equations to determine *m* and *d_m_*:

$$m= \frac{\log\left( \frac{f_{a1}}{f_{u1}} \right)-\log\left( \frac{f_{a2}}{f_{u2}} \right)}{\log D_{1}-logD_{2}}$$

Where *D_1_* and *D_2_* are two dosages for a drug representing the two ME points, and *f_a1_/f_u1_ and f_a2_/f_u2_* are their respective effects.

and

$$D_{m}=\frac{D}{\left( \frac{f_{a}}{f_{u}} \right)^{\frac{1}{m}}}$$

In addition to this approach we can use two- to five-parametric dose response models defined in the R drc package (log-logistic *LL.2-5* models) to fit the curve *Effect ~ Dose*, which allows dose-response curves produce effects in a range from a lower threshold (*E_min_*) to a higher threshold (*E_max_*) within the full effect range from 0 to 1.

#### Combination indices, confidence intervals, and additive lines

When computing combination indices (CI) our default approach relies on previous calculations of ME points and ME statistics. We calculate CI values by combining equation 1 and equation 2 (see main manuscript):

$$CI= \frac{D_{1}}{D_{m1}\left( \frac{f_{a}}{f_{u}} \right)^{\frac{1}{m_{1}}}}+ \frac{D_{2}}{D_{m2}\left( \frac{f_{a}}{f_{u}} \right)^{\frac{1}{m_{2}}}}$$

3

This equation is used with the ME statistics from the drugs and with the random ME statistics derived in the previous step. The CI from random ME statistics are used to determine the range of the CI value.

Alternatively, when LL.* models are used, computation of CI values can be performed by two different approaches:

1) The default approach is to sample a pair of points, which we call the ‘median effect points’ (ME points), and compute ME statistics from these. The median effect points are chosen close to a target range by finding the dosages that are predicted to have an effect above and below 15% of the effect range centred on the target value. Care is taken to not choose ME points outside the dosage range of the dose-response data for that drug; instead the ME points are taken close to the extreme but inside the range of measured dose-responses, to avoid extrapolation of the dose-response curve. The CI value is then computed from equation 3.

2) When a user chooses to compute dose-response relationships from LL.* models instead of the median effect equation, CI values can either be obtained from the fit directly with the equation for combination index, or via median effect points from the median effect equation (equation 1 and equation 2 respectively, Supplementary Information). Median effect points are pairs of points used to estimate the m and Dm, and are derived from the model fit around the effect range in question (see Supplementary Information for more details).

When CI values are computed directly from LL.* model fits care should be taken to only compute CI values for drug combination doses that are complemented with single drug doses eliciting a larger effect, to avoid extrapolating drug response relationships far from experimentally tested doses. In the case that a single drug cannot produce the same effect level as observed for the combination equation 1 will use a dose (Dx) value approaching infinity in computing the CI value, effectively reducing the D/(Dx) fraction to zero. If neither drug in a drug combination can produce the effect of the combination the CI value is set to zero. A CI value of zero thus indicates a strong synergy, but does not allow further quantifying the combination effect.

To obtain confidence intervals for our calculations we use the dose-response models created (least squares or LL.*) to estimate randomly new pairs of ME points from the predictive distribution, using a confidence level of 95% (see pseudocode below). These random ME points give rise to random ME statistics that are used in the next phase for confidence intervals and random additive lines.

In addition to computing the CI, the ME statistics are used to determine the ‘additive line’ which is the dose-response curve that a combination would have if the drugs show no synergy or antagonism, i.e. their effects are additive and CI equals 1. The additive line is computed point wise using the following formula

$$D_{1,2}= \frac{D_{1}+D_{2}}{\frac{D_{1}}{D_{m1} {(\frac{f_{a}}{f_{u}})}^{\frac{1}{m_{1}}}}+\frac{D_{2}}{D_{m2} {(\frac{f_{a}}{f_{u}})}^{\frac{1}{m_{2}}}}}$$

Where D_1,2_ is the combination dose.

Alternatively, for LL.* models the *D*_1,2_ is computed following the formula,

$$D_{1,2}= \frac{D_{1}+D_{2}}{\frac{D_{1}}{\left( D_{x} \right)_{1}}+\frac{D_{2}}{\left( D_{x} \right)_{2}}}$$

where *(D_x_)_1_* and *(D_x_)_2_* are found from the dose-response model.

#### Pseudocode for the generation of confidence bands

function CombinationIndex(

blue_doses, blue_effects,

red_doses, red_effects,

blue_combination_dose, red_combination_dose,

combination_effect, model_type, direct_CI)

{

blue_model = fit(blue_doses, blue_effects, model_type, combination_effect)

red_model = fit(red_doses, red_effects, model_type, combination_effect)

blue_m, blue_dm = GET ME-points FROM blue_model

red_m, red_dm = GET ME-points FROM red_model

IF direct_CI THEN

CI_value = calculateDirectCI(

blue_combination_dose, red_combination_dose,

effect, blue_model, red_model)

ELSE

CI_value = calculateCI(

blue_dose, blue_m, blue_dm,

red_dose, red_m, red_dm,

effect)

FI

additive_model = additiveCurve(

blue_combination_dose, blue_m, blue_dm,

red_combination_dose, red_m, red_dm)

CREATE ARRAY random_CI_values

CREATE ARRAY random_additive_models

FOREACH (rblue_m, rblue_dm = GET random-ME-points FROM blue_model)

FOREACH (rred_m, rred_dm = GET random-ME-points FROM red_model)

rCI_value = calculateCI(

blue_dose, rblue_m, rblue_dm,

red_dose, rred_m, rred_dm,

effect)

APPEND random_CI_values WITH rCI_value

radd_mod = additiveCurve(

blue_dose, rblue_m, rblue_dm,

red_dose, rred_m, rred_dm)

APPEND random_additive_models WITH radd_mod

ENDFOREACH

ENDFOREACH

CI_interval = [min(random_CI_values), max(random_CI_values)]

CREATE PLOT with all these data

}

function fit(doses, effects, model_type, median_effect_point)

{

EFFECT_GAP = 0.15

MAX_EFFECT = 0.95

MIN_EFFECT = 0.05

NO_RANDOM_ME_POINTS = 25

# Make median_effect_point be within the observed ranges of

# effects for the drug, and take two effects values close to

# it (inside the 0.05 and 0.95 range)

IF model_type == 'least_squares' THEN

median_effect_point = 0.5

ELSE

IF median_effect_point > max(doses) THEN

median_effect_point = max(doses) * 0.9

FI

IF median_effect_point < min(doses) THEN

median_effect_point = min(doses) * 1.1

FI

END

total_range = max(doses) - min(doses)

effect1 = median_effect_point - EFFECT_GAP * total_range

effect2 = median_effect_point + EFFECT_GAP * total_range

effect1 = MIN_EFFECT if effect1 < MIN_EFFECT

effect2 = MAX_EFFECT if effect2 > MAX_EFFECT

IF model_type == "least_squares" THEN

fit_model = FIT least squares FOR log(Effect)/log(1-Effec) ~ log(Dose)

ELSE

fit_model = FIT drc(model_type) FOR Effect ~ Dose

FI

dose1 = FIND dose that elicits effect1 FROM fit_model

dose2 = FIND dose that elicits effect2 FROM fit_model

confidence_interval1 = FIND confidence interval AT dose1 FROM fit_model

confidence_interval2 = FIND confidence interval AT dose2 FROM fit_model

m, dm = computeMDM(dose1, effect1, dose2, effect2)

CREATE ARRAY random_m_dm

FOR 1 to NO_RANDOM_ME_POINTS DO

reffect1 = random value FROM confidence_interval1

reffect2 = random value FROM confidence_interval2

rm, rdm = computeMDM(dose1, reffect1, dose2, reffect2)

APPEND random_m_dm WITH rm, rdm

ENFOR

RETURN(m, dm, fit_model, random_m_dm)

}

# The functions computeMDM, calculateCI, and additiveCurve are derived

# directly from the equations in ME theory. The function calculateDirectCI

# is derived from the Loewe additivity model
